# Evaluation of a Hub-and-Spoke Model to Enhance Healthcare Professionals’ Practice of Antimicrobial Stewardship (AMS) Programmes in the Volta Region of Ghana

**DOI:** 10.3390/antibiotics14070672

**Published:** 2025-07-02

**Authors:** Mairead McErlean, Eneyi Kpokiri, Preet Panesar, Emily E. Cooper, Jonathan Jato, Emmanuel Orman, Hayford Odoi, Araba Hutton-Nyameaye, Samuel O. Somuah, Isaac Folitse, Thelma A. Aku, Inemesit O. Ben, Melissa Farragher, Leila Hail, Cornelius C. Dodoo, Yogini H. Jani

**Affiliations:** 1 Centre for Medicines Optimisation Research & Education—CMORE, University College London Hospitals NHS Foundation Trust, 250 Euston Road, London NW1 2PG, UK; mairead.mcerlean@nhs.net (M.M.); preet.panesar@nhs.net (P.P.); emily.cooper48@nhs.net (E.E.C.); 2 Clinical Research Department, Faculty of Infectious & Tropical Diseases, London School of Hygiene and Tropical Medicine, Keppel Street, London WC1E 7HT, UK; eneyi.kpokiri@lshtm.ac.uk; 3 School of Pharmacy, University of Health and Allied Sciences, Ho PMB 31, Ghana; jjato@uhas.edu.gh (J.J.); eorman@uhas.edu.gh (E.O.); hodoi@uhas.edu.gh (H.O.); aharaba@uhas.edu.gh (A.H.-N.); sosomuah@uhas.edu.gh (S.O.S.); talalbila@uhas.edu.gh (T.A.A.); ioben@uhas.edu.gh (I.O.B.); cdodoo@uhas.edu.gh (C.C.D.); 4 Pharmacy Directorate, Ho Teaching Hospital, Ho P.O. Box MA 374, Ghana; ifolitse@yahoo.com; 5 Infection Prevention and Control Department, University College London Hospitals NHS Foundation Trust, 5th Floor Central, 250 Euston Road, London NW1 2PG, UK; melissa.farragher@nhs.net (M.F.); leila.hail@nhs.net (L.H.); 6 School of Pharmacy, University College London, 29–39 Brunswick Square, London WC1N 1AX, UK

**Keywords:** antimicrobial stewardship, hub-and-spoke model, antimicrobial resistance

## Abstract

**Background**: Antimicrobial resistance (AMR) poses a critical global health challenge, particularly in resource-limited settings. A hub-and-spoke model, decentralising expertise and distributing resources to peripheral facilities, has been proposed as a strategy to enhance the antimicrobial stewardship (AMS) capacity in low- and middle-income countries. **Aim**: This study sought to understand healthcare professionals’ experiences of a hub-and-spoke AMS model in the Volta Region of Ghana and its influence on clinical practice, leadership, and collaborative endeavours to address AMR. **Methods**: A qualitative descriptive design was adopted. In-depth interviews were conducted with 11 healthcare professionals who participated in the AMS program. Thematic analysis was used to identify key themes related to the knowledge and skills that were gained, clinical and leadership practice changes, capacity building, and challenges. **Results**: Participants reported an increased awareness of AMR, particularly regarding the scale and clinical implications of antimicrobial misuse. The clinical practice improvements included more judicious prescribing and enhanced adherence to infection prevention and control measures. Many respondents highlighted stronger leadership skills and a commitment to capacity building through AMS committees, multidisciplinary collaboration, and cross-organisational knowledge exchange. Despite resource constraints and logistical hurdles, participants expressed optimism, citing data-driven approaches such as point prevalence surveys to track progress and inform policy. Engagement with hospital management and public outreach were viewed as essential to sustaining AMS efforts and curbing over-the-counter antibiotic misuse. **Conclusions**: The hub-and-spoke model caused observable improvements in AMS knowledge, clinical practice, and leadership capacity among healthcare professionals in Ghana. While challenges remain, particularly in securing sustainable resources and shifting community behaviours, these findings underscore the potential of network-based programs to catalyse systemic changes in tackling AMR. Future research should explore long-term outcomes and strategies for embedding AMS practices more deeply within healthcare systems and communities.

## 1. Introduction

Antimicrobial resistance (AMR) is a global public health threat that contributes to excess morbidity and mortality and increases associated healthcare costs. A recent analysis estimated that bacterial AMR was associated with 5 million excess deaths worldwide [1]. The greatest increase in antimicrobial consumption has been observed in low- and middle-income countries (LMICs) [2]. As a result, antimicrobial stewardship (AMS) programmes have received increasing resource and policy focus in LMICs.

In Ghana, multiple studies on antimicrobial use have highlighted the variations in and trends of clinical practice over time [3,4,5,6,7,8,9]. These findings emphasise the need for sustained stewardship interventions, both at a national and local level. The Volta Region in southeastern Ghana includes both urban and rural districts with variable access to healthcare infrastructure. Community pharmacies play a significant role in medicine provision, including the provision of antimicrobials, which poses both opportunities and challenges for AMS implementation.

The Commonwealth Partnerships for Antimicrobial Stewardship (CwPAMS) programme, funded by the UK aid Fleming Fund and managed by the Commonwealth Pharmacists Association (CPA) and Tropical Health and Education Trust (THET), supports global partnerships to strengthen AMS systems in LMICs [10]. Since 2019, a partnership between the University College London Hospitals NHS Foundation Trust (UCLH), UK, the University of Health and Allied Sciences (UHAS), Ho, Ghana and the Ho Teaching Hospital (HTH), Ghana, has supported AMS capacity building in the Volta Region of Ghana. Initially focused on the HTH, the programme later adopted a hub-and-spoke model to decentralise stewardship activities and promote knowledge exchange with four smaller district hospitals. A hub-and-spoke model, which involves a central hub institution supporting peripheral spoke sites through shared expertise, capacity building, and coordinated activities, has been successfully applied in AMS initiatives across low- and middle-income countries to improve stewardship outcomes and enable system-wide collaboration [11].

This paper explores healthcare professionals’ experiences with a hub-and-spoke AMS model in Ghana and evaluates its influence on clinical practices, leadership development, and collaborative strategies to combat AMR.

## 2. Results

### 2.1. Summary of Participant Characteristics

In October 2024, a total of 11 participants were interviewed for this study (Table 1). The participants were from five healthcare institutions in the Volta Region and one academic institution. Two participants were recruited from each study site, along with one participant from the coordinating university. The roles of the participants included prescribers and non-prescribers such as pharmacists, nurses, physician assistants, and administrators. Most participants were members of their facility’s AMS committee. The duration of involvement of the participants in the AMS programme ranged from one week to over five years, reflecting a breadth of experience across tertiary and district-level facilities.

### 2.2. Emerging Themes from Qualitative Interviews

**Figure 1:**
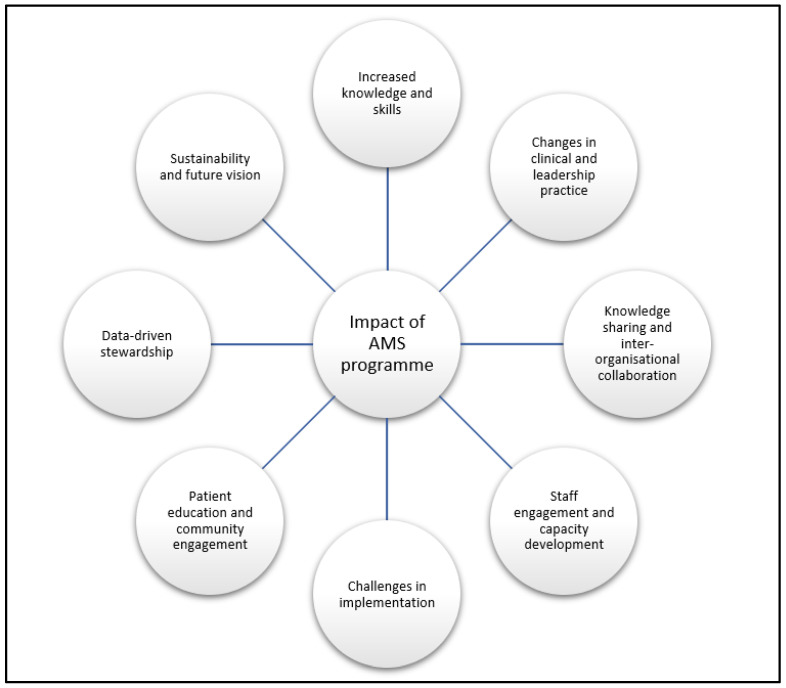
Emerging themes of the impact of a hub-and-spoke model AMS programme.

Thematic analysis of the interviews identified eight major themes that reflected the impact of the AMS programme (Figure 1).

#### 2.2.1. Increased Knowledge and Skills

Participants reported significantly improved understanding of AMR, its clinical implications, and the importance of infection prevention and control (IPC). For many, the programme addressed gaps in prior knowledge and enhanced awareness of practices such as hand hygiene and the appropriate disposal of unused antimicrobials.

“Yes, I knew antimicrobials have resistance; I never knew you had quite a huge number of resistance to antimicrobials prior to this engagement.”

(Nurse, Hub site)

“I was a little bit oblivious to the danger we are facing currently with regards to antimicrobial resistance... I actually knew that yes... some bacteria are resistant to some antibiotics, but I always had the impression that we could just switch to a different one.”

(Medical Officer, Spoke Site)

“I’m hoping that especially with this current training, that there would be a reduction in hospital acquired infections.”

(Administrator, Spoke site)

#### 2.2.2. Changes in Clinical and Leadership Practice

Participants described shifts towards more judicious prescribing, greater use of diagnostics (e.g., culture and sensitivity tests), and enhanced adherence to guidelines. Leadership development was also highlighted, with participants gaining confidence in guiding colleagues and contributing to AMS committees and institutional decision-making.

“I make sure the patient really needs an antimicrobial before I prescribe. Meanwhile, at first, I mean, I was a little bit liberal sometimes.”

(Medical Officer, Spoke site)

“The need for targeted therapy has increased and the request for culture sensitivity prior to starting some antibiotics or to inform the change of antimicrobial has changed.”

(Nurse, Hub site)

“And most of us on the committee are leaders in a way, how are you able to manage your subordinates to be able to send out information for them to grasp, and also how to know the type of leader that you are and what kind of leadership can be used in what circumstance. So yes, it has been very, very, very enlightening.”

(Physician Assistant, Hub site)

#### 2.2.3. Knowledge Sharing and Inter-Organisational Collaboration

Participants frequently shared the knowledge gained through workshops and collaborated across departments and facilities. AMS teams were established within institutions, fostering cross-disciplinary and cross-organisational knowledge exchange.

“We formed an antimicrobial stewardship team within my facility, so we hold meetings, we meet with hospital managers, we meet with our colleagues on all the various topics to present what we learn. We do all the workshops for them to be able to influence their practice and then decision-making and other things, so we are supposed to be something like agents of antimicrobial stewardship.”

(Medical Officer, Spoke site)

“We take ideas from each other and after the workshops also we try to link up with other facilities to see what we are doing right and what we aren’t and what they are also doing and what we are also doing …We call ourselves the sister facilities, so we get knowledge from each other.”

(Nurse, Spoke site)

#### 2.2.4. Staff Engagement and Capacity Development

Participants reported that the programme boosted motivation and professional development. Participants cited enhanced skills in project management, public engagement, and manuscript writing. Some assumed leadership roles within their facilities or broader AMS networks.

“I’ve benefited... a lot from it in terms of project management, publication writing, manuscript writing, public engagement, moderating programmes because I’ve been MC for a number of times and I’m a lead for the medicine disposal, the take back programme.”

(Pharmacist, Academic Institute)

“Being involved in the leadership planning has also boosted my abilities and capabilities of leadership.”

(Nurse, Hub site)

#### 2.2.5. Challenges in Implementation

Key barriers included limited funding, staff shortages, competing priorities, and a lack of laboratory equipment. Resistance to behavioural change among healthcare staff and patients also emerged as a major challenge.

“...Most of the time you present certain things [to management] that need to be done, and then the feedback you get is there’s no money; you’ve not budgeted for it.”

(Medical Officer, Spoke site)

“Currently my facility doesn’t have a culture and sensitivity test machine, so we do it outside the facility, which is a challenge.”

(Nurse, Spoke site)

“In my facility, for instance, we don’t have many doctors. So yes, so when it’s time for a workshop, I often really struggle to get someone to cover for me while I’m away for the three hours of the workshop. But I still try to attend and learn a lot and then come back to impact.”

(Medical Officer, Spoke site)

“It’s a challenge; the culture of Ghanaians, most of us, when we are sick, you go to the drugstore first... And you take the medications given to you by the drugstore attendant and you are fine. There’s no need for the hospital. So, most of the time, the community usually dispense antimicrobials anyhow in terms of the pharmacies and because they’re making money, there’s no motivation to stop. So that’s one challenge that I foresee that I’m going to face.”

(Medical Officer, Spoke site)

#### 2.2.6. Patient Education and Community Engagement

Public outreach through media, churches, and interpersonal networks was seen as key to reducing inappropriate antibiotic use. Participants promoted responsible self-medication behaviours and encouraged community pharmacy involvement in stewardship efforts.

“We do out-of-hospital public engagements as well. So radio station, TV stations, yes.”

(Nurse, Hub site)

#### 2.2.7. Data-Driven Stewardship

The Global Point Prevalence Survey (GPPS) was widely valued as a tool for assessing antimicrobial use and monitoring improvements. Sharing GPPS findings with prescribers and management was reported to influence prescribing practices and institutional policy.

“...From the very first GPPS we did, and then the subsequent ones, we’ve realised some change, but we actually reported that in our paper. And we showed the prescribing patterns in a teaching hospital in Ghana. So we did it in, I think, January and then we did in July. So we’re able to compare and realise that there was a positive change in the prescribing patterns.”

(Pharmacist, Academic Institute)

“The data from the from the GPPS. Yes, is being presented to management and... the prescribers. And after the GPPS summary, most of the prescribers are also on board. I mean the data is gathered with some of the prescribers. So that also informs on their prescription of antimicrobial, especially with regard to doing targeted treatment I mean, yes.”

(Physician Assistant, Hub site)

#### 2.2.8. Sustainability and Future Vision

Participants highlighted the importance of embedding AMS practices into routine institutional activities to ensure long-term sustainability. This included the establishment of ward-level champions, continued training for new staff, and securing management support and resources.

“…we are putting plans in place... to be able to make sure that it doesn’t end this year or next year like it becomes embedded in... the hospitals, activities. So, we are looking at having an AMS team on the wards or like various representatives on the units so that they are like champions... when new staff come to the facility, there’s training for them. And the push so the prescribers are able to prescribe based on the guidelines.”

(Administrator, Spoke site)

“…my expectation is by the end of this project, we’re able to have a solid team; we get more departments and people on board to support, especially if we get the support from management because some of these things will need funding. We’ll get the support from management.”

(Nurse, Spoke site)

## 3. Discussion

### 3.1. Summary of Main Findings

This qualitative evaluation of a hub-and-spoke AMS model in Ghana’s Volta Region revealed several important findings. The participants consistently described improved understanding and heightened awareness of AMR, IPC, and responsible prescribing practices. These improvements were reflected in reported changes to clinical decision-making, an increased use of diagnostics, the formation of AMS committees, and strengthened leadership roles. Cross-disciplinary collaboration and community engagement were also notable, alongside challenges that included limited resources and the need for long-term sustainability. Many participants also valued data-driven monitoring tools such as the GPPS to assess prescribing trends and guide targeted interventions.

### 3.2. Comparison with Literature

The increase in AMS knowledge and improved practices reported by participants aligns with World Health Organisation (WHO) recommendations which emphasise ongoing training and the use of evidence-based protocols in LMICs to combat AMR [12]. These findings are further supported by a recent scoping review that identified staff education, training, and multidisciplinary teamwork as key facilitators in AMS implementation across LMIC hospital settings [13]. In the Volta Region, education was not only beneficial to prescribers but also to non-prescribing staff who engaged in IPC practices, committee work, and interprofessional communication.

The observed leadership development and implementation of structured AMS roles echoed findings from other LMICs where strong institutional support, clear governance, and managerial engagement are consistently associated with successful stewardship interventions [14,15]. The participants’ ability to lead AMS initiatives and influence change within their facilities illustrates how decentralised models can empower healthcare workers and strengthen stewardship capacity across varied institutional contexts.

Multidisciplinary collaboration was a notable strength of the model, with several participants describing interprofessional teamwork and cross-institutional learning. These findings are consistent with a recent qualitative study across LMICs which highlighted the importance of multi-professional perspectives and local adaptation in effective AMS programme implementation [16].

Community engagement and patient education were also critical, with participants noting efforts to improve public awareness through outreach and informal education. These activities align with global calls for AMS to move beyond the hospital to address public awareness and behavioural change [17]. However, over-the-counter access to antibiotics was frequently cited as a persistent barrier. This is consistent with wider evidence that self-medication is prevalent in LMICs, driven by poor regulation, limited access to healthcare, and insufficient public knowledge [18]. Given its prominence in participant interviews, addressing community-level antibiotic misuse remains an urgent target for AMS strategies in similar contexts.

The positive reception of the GPPS as a structured, data-driven tool for improving prescribing supports the literature, emphasising the role of surveillance and feedback mechanisms in antimicrobial use [19]. However, maintaining consistent data collection in resource-constrained environments can be difficult, especially when they are reliant on external support or short-term projects.

Participants also acknowledged that sustaining AMS efforts depends on long-term integration into routine practice, with institutional commitment and funding being crucial. This is supported by previous programmes of work which advocate embedding AMS into national health systems and hospital infrastructures to promote programme continuity beyond externally funded initiatives [15].

### 3.3. Limitations

This study included a modest number of participants (*n* = 11), which may limit its generalisability. Although the interviews provided detailed insights, self-reporting could have introduced bias, and the short data collection period may not reflect long-term sustainability or institutional change. Nevertheless, the findings offer valuable contributions to understanding AMS implementation in low-resource settings and provide practical insights for scaling such models.

## 4. Materials and Methods

This qualitative study was conducted in the Volta region of Ghana as part of an AMS capacity-building programme implemented through a partnership between the University of Health and Allied Sciences (UHAS), Ho Teaching Hospital (HTH), and University College London Hospitals NHS Foundation Trust (UCLH). The partnership was established in 2019 through the Commonwealth Partnerships for Antimicrobial Stewardship (CwPAMS) programme. The hub-and-spoke model was formally launched in 2023 following the approval of a 2-year AMS action plan co-developed by UHAS, HTH, and UCLH. The model enabled structured coordination between the hub-and-spoke sites through virtual and in-person engagement, with each spoke site establishing its own AMS committee and participating in activities including ward rounds, audits, surveillance, and awareness campaigns.

For this evaluation of a hub-and-spoke AMS model, HTH was the hub site and the four spoke sites were Ho Municipal Hospital, Margret Marquart Catholic Hospital, Volta Regional Hospital, and Ketu South Municipal. Eleven participants were purposively selected to represent all five hospitals and the coordinating university. This number was guided by practical considerations including participant availability and the diversity of professional roles. It included two participants from each hospital and one academic lead from the university. All interviewees attended the AMS workshop where recruitment occurred. No participants declined, although some were unavailable during the interview window. Informed consent was obtained, and semi-structured interviews were conducted by a UCLH researcher (MM, a female research pharmacist) via Microsoft Teams in October 2024. Interviews lasted 20–30 min and followed a topic guide developed by the study team. Audio recordings were transcribed verbatim.

Thematic analysis was undertaken using a combination of inductive and deductive approaches guided by the theoretical domains framework (TDF) [20]. Initial coding was conducted by MM and independently reviewed by two researchers (YHJ, an experienced female clinical academic pharmacist, and EK, a female assistant professor). A final codebook was agreed upon by team consensus. Coding was conducted manually.

Ethical approval (GHS-ERC: 024/08/23) was obtained from the Ghana Health Service Ethics Review Committee. Data were anonymised and stored in compliance with GDPR and UCLH trust policy.

To strengthen validity, triangulation was achieved through multiple coders and cross-checking of findings. The interviewer’s external position helped reduce bias and reflexivity was maintained through ongoing team discussions. This study was reported in line with the Consolidated Criteria for Reporting Qualitative Research (COREQ) checklist [21].

## 5. Conclusions

The hub-and-spoke model implemented in Ghana’s Volta Region was reported to have contributed to improved AMS knowledge, clinical practice, and leadership capacity among healthcare professionals. Despite ongoing challenges, particularly regarding resource constraints and public behaviour, this model demonstrates the potential of network-based programmes to tackle AMR in LMICs. Future research should examine long-term sustainability and strategies for integrating AMS more fully into routine health system structures and community engagement efforts.

## Figures and Tables

**Table 1:** Interview participant details including place of work, profession, and duration of involvement in the programme.

Participant Number	Organisation	Profession	Member of AMS Committee	Duration Involved in Project
1	Ho Teaching Hospital	Nurse	Yes	5 years
2	Ho Teaching Hospital	Physician Assistant	Yes	2 years
3	Ho Municipal Hospital	Pharmacist	Yes	6–12 months
4	Ho Municipal Hospital	Administrator	Yes	6–12 months
5	Ketu South Municipal Hospital	Pharmacist	Yes	6–12 months
6	Ketu South Municipal Hospital	Nurse	Yes	6–12 months
7	Margret Marquart Catholic Hospital	Administrator	Yes	1–6 months
8	Margret Marquart Catholic Hospital	Medical Officer	Yes	6–12 months
9	Volta Regional Hospital	Administrator	No	<1 month
10	Volta Regional Hospital	Nurse	No	6–12 months
11	University of Health and Allied Sciences	Pharmacist	Yes	6 years

## Data Availability

The data presented in this study are available on request from the corresponding author due to privacy restrictions.
